# Clara cell adhesion and migration to extracellular matrix

**DOI:** 10.1186/1465-9921-9-1

**Published:** 2008-01-07

**Authors:** Jeffrey J Atkinson, Tracy L Adair-Kirk, Diane G Kelley, Daphne deMello, Robert M Senior

**Affiliations:** 1 Department of Internal Medicine, Pulmonary and Critical Care Division, Washington University School of Medicine, St. Louis, MO, USA; 2 Department of Cell Biology and Physiology, Washington University School of Medicine, St. Louis, MO, USA; 3 Department of Pathology, Phoenix Children's Hospital, Phoenix, AZ, USA

## Abstract

**Background:**

Clara cells are the epithelial progenitor cell of the small airways, a location known to be important in many lung disorders. Although migration of alveolar type II and bronchiolar ciliated epithelial cells has been examined, the migratory response of Clara cells has received little attention.

**Methods:**

Using a modification of existing procedures for Clara cell isolation, we examined mouse Clara cells and a mouse Clara-like cell line (C22) for adhesion to and migration toward matrix substrate gradients, to establish the nature and integrin dependence of migration in Clara cells.

**Results:**

We observed that Clara cells adhere preferentially to fibronectin (Fn) and type I collagen (Col I) similar to previous reports. Migration of Clara cells can be directed by a fixed gradient of matrix substrates (haptotaxis). Migration of the C22 cell line was similar to the Clara cells so integrin dependence of migration was evaluated with this cell line. As determined by competition with an RGD containing-peptide, migration of C22 cells toward Fn and laminin (Lm) 511 (formerly laminin 10) was significantly RGD integrin dependent, but migration toward Col I was RGD integrin independent, suggesting that Clara cells utilize different receptors for these different matrices.

**Conclusion:**

Thus, Clara cells resemble alveolar type II and bronchiolar ciliated epithelial cells by showing integrin mediated pro-migratory changes to extracellular matrix components that are present in tissues after injury.

## Background

Clara cells are epithelial cells on the luminal surface of airways with a dome shaped cytoplasmic protrusion and no cilia [1,2]. In addition to their secretory and xenobiotic roles [3,4], Clara cells are the progenitor cell in small airways [5]. After airway injury, Clara cells in stem cell niches proliferate and migrate to replenish the injured terminally differentiated epithelial cells [6]. In fact, after alveolar injury, Clara cells can be seen in the alveolus (alveolar bronchiolization), suggesting the response of the terminal airway epithelium to alveolar injury exceeds the rate of alveolar epithelial cell repair [7,8].

Epithelial repair requires a complex series of steps including cell spreading and/or migration over the exposed interstitial matrix and provisional matrix to form intact tight junctions (restitution), and replenishment of the initial cell density by proliferation and redistribution of differentiated epithelial cells over the provisional matrix (reconstitution). The response of epithelial cells to different matrix components is of interest, as the provisional matrix, which is generated after injury, contains new epitopes that can influence epithelial cell commitment to migration [9-15]. The addition of matrix molecules can induce non-directional pro-migratory behaviour (chemokinesis) in epithelial cells, but some epithelial cells will migrate towards soluble gradients of substrate (chemotaxis) or affixed substrates which present a gradient of adhesion sites (haptotaxis). Large airway ciliated cells and alveolar type II cells both show haptotactic migration toward the provisional matrix molecule fibronectin [9,12-14], but the directed migration of Clara cells has only been reported in mixed cell preparations where the percentage of Clara cells migrating was not defined [18].

At steady state, the basement membrane is composed of two parallel sheets of laminin (Lm) and collagen (Col). In the adult lung, the topmost layer is composed primarily of Lm 332 (formerly laminin-5) and Lm 511 while the lower layer is Col IV [16]. The underlying interstitial matrix contains fibroblasts in a fibrillar Col (I and III) matrix. Following disruption of the epithelial cell layer and basement membrane, a provisional matrix rich in fibronectin (Fn) is formed. Provisional matrix molecules contain multiple RGD (arginine-glycine-aspartic acid) epitopes that are not present within the steady state matrix [17]. This short peptide sequence provides sites for interaction of epithelial cell surface receptors (integrins) during cell migration [11]. Migrating cells must alter surface receptors for adhesion and traction across the provisional matrix. The matrix-associated receptors in airway epithelial cells during steady state are predominantly collagen and laminin binding integrins (α2β1, α3β1 and α6β4), that do not bind classic RGD epitopes [18]. After injury bronchiolar epithelial cells express α5 and αv containing integrins (fibronectin and vitronectin receptors) at the wound edge [18]. Clara cells express α5, α6, αv, β1 and β4, but do not express β3 integrin the typical partner for αv [19].

The evaluation of Clara cell migration has been hampered by their overall low abundance in human lungs and restriction to the terminal airway. Although Clara cells are much more abundant in rodents, especially in the terminal airways [20], primary culture is complicated by relatively impure yields and a rapid loss of phenotypic characteristics in culture [19,21-25]. Until recently, immortalized airway epithelial cell lines produced only minimal amounts of Clara cell specific proteins and lacked contact inhibition [26]. To overcome these difficulties the C22 cell line was developed by isolating Clara cells from the Imortomouse™ [27]. All cells from the Imortomouse™ harbour a transgenic, temperature-sensitive, MHC-driven, large-T antigen. Although these cells secrete minimal Clara cell secretory protein (CC10), they make CC10 mRNA and surfactant protein B but not C, which defines them as Clara-like cells [27]. The C22 cells display many of the characteristics of mature Clara cells, including growth in monolayers and formation of tight junctions [27].

The objectives of this study were to devise a reproducible procedure for murine Clara cell isolation, to evaluate Clara cell migration to several matrix substrates relevant to wound repair, and to compare the C22 cell line to Clara cells in adhesion and migration assays. After determination that C22 cells were similar to freshly isolated Clara cells in assays of migration, we examined the role of RGD-binding integrins on C22 cell migration.

## Methods

### Clara Cell Isolation

Clara cells were isolated from Swiss Webster mice (Taconic Farms, Germantown, NY) using a protocol modified from Corti, et. al [28]. Mice were injected i.p. with heparin (250 u/mouse) and killed by CO_2 _narcosis. After transection of the dorsal vessels, the right ventricle was canulated and the lungs were flushed with cold phosphate buffered saline (PBS) until white (~10 ml) by directing the catheter towards the main pulmonary artery. The trachea was canulated with a 22 g catheter and 0.5 ml of 1% low melting temperature agarose (Gibco, Grand Island, NY) in PBS was instilled and the lungs were placed on ice for 2 min. Then 0.5 ml of 0.25% bovine pancreatic trypsin (Sigma, St. Louis, MO, T-8003) in Hank's balanced salt solution (without Ca^++ ^or Mg^++^) was instilled, the trachea was secured with a suture and the excised lungs were incubated at 37°C for 10 min. Using a scalpel and scissors, lung tissue was teased away from the large airways, diced to approximately 1 mm^3 ^pieces, and placed in DMEM containing 250 μg/ml of DNAse I and antibiotic/antimycotic (Cellgro, Herndon, VA) for 5 min minutes. The resulting suspension was transferred to a 50 ml conical tube, and fetal calf serum was added to achieve 10% of final volume.

To remove the non-cellular portion, the suspension was filtered through 100 and 40 μm filters sequentially and the filtrate was sedimented by centrifugation at 200 g for 10 minutes. The cell pellet was resuspended in DMEM with antibiotic/antimycotic and spun three times at 32 g for 6 min at 10°C, each time discarding the supernatant and resuspending the pellet in DMEM with antibiotic/antimycotic.

Cells were plated for 1 hr on 100 mm tissue culture dishes pre-coated with 500 μg/ml mouse IgG (Sigma) to remove fibroblasts. The non-adherent cells were collected and aliquots were stained with nitroblue tetrazolium (NBT) as described below to identify the Clara cells. The cells were resuspended in DMEM containing 2% fetal calf serum and antibiotic/antimycotic for all assays.

### NBT Staining

To identify Clara cells, NBT staining was performed. Cells were affixed to glass slides using a Shandon cytospin (5 min, 500 rpm). After air drying, cells were fixed in 10% phosphate buffered formalin for 30 sec. Slides were incubated in a fresh solution of 0.1% NBT (Sigma, N-6876) and 0.1% NADPH (Sigma, N1630) in PBS with 25 mM HEPES for 10 min at 37°C. Then slides were rinsed in distilled water and counterstained with methyl green. Only Clara cells stain dark purple (see additional file 1 panel A). The brief formalin fixation is necessary as some non-Clara cells demonstrate weak purple staining.

### Immunostaining for Cell Specific Markers

Commercial antibodies and concentrations were as follows: 1:1000 mouse anti-pan cytokeratin mixture (C2562, Sigma, St. Louis), 1:5000 rabbit anti-pro-SP-C (Chemicon, Temecula, CA), 1:500 mouse anti-β-tubulin-IV (Biogenex, SanRamon, CA) and 1:200 mouse anti-vimentin (Sigma, St. Louis, MO). Donkey anti-CC10 antibody was a gift from Dr. G Singh (Pittsburgh VA Medical Center) and used at 1:10,000. Mouse and rabbit IgG to control for nonspecific binding were from Biocare Medical (Concord, CA). FITC and TRITC-conjugated secondary antibodies were used at a 1:200 concentration (Jackson Labs, Bar Harbor, ME). Mounting media containing DAPI (Vector Labs, Burlingame, CA) was used for immunofluorescent staining.

### Culture of C22 cells

C22 cells were developed as described [27]. They are a conditionally immortalized cell line isolated from a C57Bl/10 × CBA hybrid strain mouse harbouring a temperature sensitive, MHC driven large T antigen transgene (Immortomouse, Charles River Labs). All cells have undergone less than 30 passages. Cells were grown in permissive conditions (33°C and proliferative media) prior to experiments then transferred to non-permissive conditions (39°C and differentiation media) 24 hrs prior to any experiments to inactivate the large T-antigen. Differentiation media is as defined [27] and proliferative media contains the same supplements plus interferon γ (100 U/ml) to induce the expression of the MHC driven large T-antigen.

### Coating of Surfaces with Matrix Substrates

Bovine Fn (Sigma-Aldrich), mouse laminin 1 (Lm 111) (Sigma-Aldrich), mouse Col IV (Chemicon), human placental laminin 10 (Lm 511) (Chemicon), and rat tail Col I (BD Biosciences, Bedford, MA) were diluted to 50 mg/ml in PBS. For Lm 332 the conditioned media of rat bladder carcinoma cells (804 G, gift of Dr. G. Plopper, Rensselaer Polytechnic Institute, NY) in DMEM (without serum) was used undiluted. 804 G cells make a matrix that is predominantly Lm 332 and the supernatant is enriched for the processed form of Lm 332 [29,30]. Chamber slides were coated with the matrix substrates by incubation of the slides with matrix solution for 1 hr at room temperature. Slides were washed 3 times with PBS and air-dried. The undersurfaces of the 8 μm polycarbonate membranes (Neuro Probe Inc, Gaithersburg, MD) used in haptotaxis experiments were coated with matrix substrates by floating the membrane on the matrix solutions for 1 hr at room temperature.

### Adhesion Assays

Clara cells were plated on uncoated glass or chamber slides coated with matrix substrates. The cells were incubated for 18 hrs in 5% CO_2 _at 37°C, then the slides were gently washed with PBS three times to remove non-adherent cells and mounted with a DAPI counterstain. Cells were stained for CC10 to determine the identity of adherent cells. The number of adherent Clara cells was counted in five high power fields (hpf, 200×) per slide. Results represent the average number of cells per hpf from at least 3 independent isolations +/- SEM. For studies using C22 cells, procedures were similar except the cells were harvested using 0.05% trypsin/53 mM EDTA for 3 minutes and incubation for adhesion was only 30 min.

### Proliferation Assays

Clara cells were plated on uncoated glass or chamber slides coated with matrix substrates. After 18 hrs at 37°C in 5% CO_2_, unattached cells were removed by gentle washing with PBS. The media was replaced with DMEM containing 10% fetal calf serum and 10 μg/ml bromodeoxyuridine (BrdU, Sigma-Aldrich) and cells were incubated for an additional 24 hrs. Cells were fixed with 10% phosphate buffered formalin for 10 min at room temperature and permeablized with PBS containing 0.5% Triton X-100. BrdU incorporation was evaluated by immunostaining using a 1:200 dilution of rat anti-BrdU antibody (Accurate Chemical, Westbury, NY) followed by a 1:200 dilution of TRITC-conjugated anti-rat secondary antibody. Slides were mounted with aqueous mounting media containing DAPI. The TRITC-positive and DAPI-positive cells were counted in five hpfs per slide. Results represent the average percentage of cells that underwent proliferation during the time period of the assay (i.e. the number of TRITC-positive cells divided by the number of DAPI-positive cells) per hpf from at least 5 independent experiments +/- SEM.

### Haptotaxis

Haptotaxis is directed migration towards an increasing gradient of adhesion sites. Typically incubation of a porous membrane with a saturating concentration of the substrate that contains the necessary epitopes will create a gradient that is greatest on the coated surface. Membranes were coated as described then placed with coated surface down in modified Boyden chambers (Neuroprobe, Gaithersburg, MD). Clara cells or trypsinized C22 cells (2 × 10^6 ^cells in 50 μl) were placed in the upper well of modified Boyden chambers. Clara cells require 2% serum for adhesion [31], so serum was added to the upper well only (serum was not added in migration experiments of C22 cells). Chambers were placed in an incubator at 37°C and 5% CO_2 _and cells were allowed to migrate for 8, 16 or 24 hrs. Cells were fixed and stained with Dif-quick (Fisher Scientific, Houston, TX), then non-migrated cells were manually removed from the upper membrane surface by gentle friction with a cotton swab. Because migration can be greater in the center than edges of wells, only the center of each well was examined. The number of migrated Clara cells was counted in one hpf, at the center of each well in twelve individual wells per experiment. Results represent the average number of cells migrated per hpf from at least three independent experiments +/- SEM (at least 10 wells per condition). In independent experiments, C22 cells were incubated with GSRGD or GSRGE peptides for 5 min at room temperature prior to addition of the cell/peptide mixture to haptotaxis chambers. The GSRGD and GSRGE peptides were a gift from Dr. R. Mecham (Washington University, St. Louis, MO) and synthesized as described [32].

### Statistical analysis

The statistical significance of all results were evaluated by performing an ANOVA test with a Scheffe posthoc analysis to account for the multiple comparisons between conditions or concentrations (SPSS 13, SPSS, Chicago, Il).

## Results

### Isolation of Mouse Clara Cells

**Figure 1:**
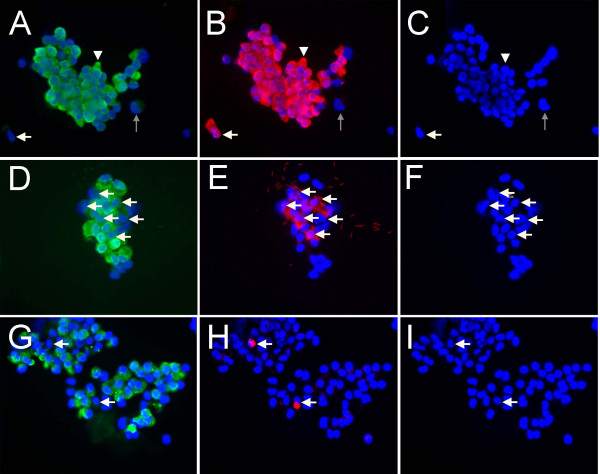
**Representative cytospin preparation of isolated cells**. **(A, D, G) **CC10 positive airway Clara cells (green) with DAPI counterstain. The same preparations were simultaneously stained for either **(B) **epithelial cytokeratins, **(E) **β-tubulin positive ciliated cells, **(H) **pro-SPC positive alveolar type II cells (all in red). The third panel in each series demonstrates the DAPI nuclear counterstain alone **(C,F,I)**. In panel **A**, the white arrowhead demonstrates a typical Clara cell, grey arrows indicate an example of a non-epithelial/non-Clara cell and white arrows indicate examples non-Clara, epithelial cells. In **D**, **E **and **F **white arrows indicate the location of 6 ciliated cells, and in **G**, **H **and **I**, white arrows indicate the location of 2 type II cells in the panels. All images at 200×.

Previous attempts to isolate mouse Clara cells have been hampered by limited purity, cell quantity, or the availability of an elutriator [23,24,33,34]. Using a modification of a mouse type II cell isolation technique [28], we have obtained an enriched Clara cell population. This technique does not require elutriation, and gives yields similar to other published methods (Table 1). Clara cells have a high level of NADPH oxidase activity that results in reduction of the soluble yellow NBT substrate to a purple formazan precipitate. Immunohistochemistry for Clara cell specific protein CC10 (Fig 1A,D,G) and NBT staining were similar (see additional file 1), so NBT percentage was used to determine the number of Clara cells for plating. Although some ciliated cells and type II cells can stain lightly with NBT, a short fixation with formalin clearly separates the dark staining Clara cells from non-Clara cells [35]. Our yield was approximately 2 × 10^5 ^Clara cells per mouse with 60–70% NBT-positive. The number of Clara cells may be an underestimate as the Clara cells tend to clump [22], and we did not alter our counts to correct for this. Greater than 95% of cells were viable by trypan blue exclusion (data not shown).

**Table 1:** Summary of Mouse Clara Cell Isolation Procedures

First Author	Date	Mouse Strain	Elutriation	Yield	% Clara	Protease	Ref
Massey, TE	1987	CD1	+	2 × 10^5^	55–65	protease I	[23]
Oreffo, VI	1990	Cba	+	5 × 10^6^	55	trypsin	[21]
Forkert, PG	1990	CD1	+	2 × 10^5^	40	protease I	[33]
Belinsky, SA	1995	A/J	+	ns	76	protease I	[22]
Hynes, DE	1999	CD1	+	ns	64	elastase	[53]
McBride, S	2000	C57Bl/6	+	ns	75	trypsin	[35]
Walker, SR	1989	BDF1	-	4 × 10^6^	88	elastase	[25]
Chichester, CH	1991	Swiss	-	2 × 10^6^	72	elastase	[34]
Corti, M	1996	C57Bl/6	-	ns	75–80	dispase	[28]
Boogaard, PJ	2000	CD1	-	1 × 10^6^	60	trypsin	[24]
Blundell, RA	2005	C3H	-	ns	ns	trypsin	[19]
Atkinson, JJ	2007	Swiss	-	2 × 10^5^	63–80	trypsin	

ns – not stated

Similar to prior publications of Clara cell isolation techniques the primary contaminating cell type was ciliated (~20–30%) (Fig. 1E, white arrows) [25,31]. Likely this is due to the clumping of isolated cells that contain some ciliated cells still adherent to Clara cells. The numbers of cells staining for β-tubulin (Fig. 1E) may be an underestimate of total ciliated lineage cells as cilia become shortened and shed during isolation. However immediately after isolation both the shed cilia and shortened cilia could be seen in cytospin preparations (Fig. 1E). Staining for the FoxJ1 protein, which is an intracellular marker for ciliated lineage cells, was less abundant in these preparations (data not shown) so β-tubulin was used. Contamination with pro-SP-C positive, alveolar type II cells was 10% (Fig. 1H, white arrows). Greater than 90% of the cells were positive for epithelial cell specific cytokeratins (Fig. 1B) and staining for vimentin was similar to the control mouse IgG (data not shown), indicating minimal contamination with fibroblasts or non-epithelial cells.

Clara cells have been demonstrated to downregulate P450 enzymes and CC10 in less than 24 hours in culture [25], but immunohistochemistry for CC10 in our studies confirmed ~60% of cells were CC10 positive after 24 hours in culture, suggesting the adherent population is enriched for Clara cells even though CC10 staining may underestimate Clara cell numbers at this time point.

### Mouse Clara Cells Demonstrate Differential Adhesion to Matrix Components

**Figure 2:**
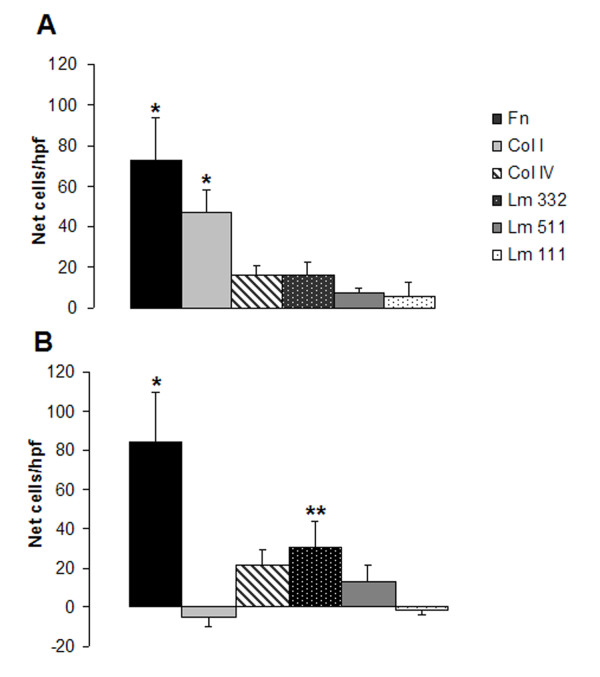
**Clara cell adhesion on matrix substrates**. **(A) **Adhesion of Clara cells to fibronectin (Fn, black), type I collagen (Col I, light grey), laminin 332 (Lm 332, black stippled), type IV collagen (Col IV, striped), laminin 511 (Lm 511, dark grey), laminin 111 (Lm 111, white stippled) slides. **(B) **C22 adhesion to Fn, Lm 332, Col IV, Lm 511, Lm 111, Col I. Results are expressed as net number of adherent cells (cells on substrate – cells on uncoated for that experiment) per high power field +/- SEM from five independent experiments. p values for comparison with uncoated control. *p < 0.001, **p < 0.05

Adhesive interactions between cells and extracellular matrix proteins are important in cellular migration and proliferation. Therefore, we examined the effects of components of the basement membrane (Lm 111, 332, 511 and Col IV) and provisional matrix (Fn, Col I) on the adhesive properties of Clara cells. Results are expressed as mean number of adherent cells per five hpfs less the mean number of adherent cells on uncoated wells in the same experiment (Net Cells/hpf +/- SEM) (Fig. 2A). Clara cells preferentially adhered to Fn (73 +/- 21) and Col I (46 +/- 11), but adhesion to Lm 332 (16 +/- 6), Col IV (16 +/- 5), Lm 511 (7 +/- 3) and Lm 111 (6 +/- 7) was not significantly above uncoated controls. These findings are consistent with prior reports that Clara cell adhesion was enhanced by Fn or Col I coating of substratum [31]. These data suggest that freshly isolated Clara cells preferentially bind to components of the provisional matrix (Fn, Col I) compared with basement membrane components (Col IV and laminins).

To confirm these findings, C22 cells were removed from plates with trypsin/EDTA to simulate conditions present during isolation. Adhesion was allowed for only 30 min and performed in the absence of serum (which was required for the Clara cells). C22 cells preferentially bound to Fn (84 +/- 29), similar to the Clara cells, but Lm 332 (30 +/- 13) demonstrated significant adhesive properties for the C22 cells while Col I (^-^5 +/- 4) was no more adhesive than the uncoated controls (Fig. 2B). The differences between isolated Clara cells and the C22 cells may represent effects of the isolation vs. the brief trypsinization of the C22 cells or an intrinsic difference in matrix adhesion receptor expression between the C22 cells and the Clara cells. Similar to the Clara cells, the C22 cells were slightly more adherent to Col IV (22 +/- 8) and Lm 511 (13 +/- 9) than Lm 111 (-1 +/- 2) (not statistically significant from each other or uncoated). Although all are basement membrane molecules, Lm 111 is not expressed by the lung except during early embryonic development [36].

### Mouse Clara Cells Proliferate in vitro Independent of Matrix Substratum

**Figure 3:**
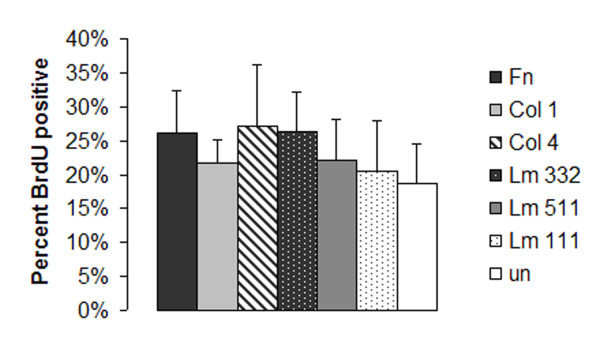
**Clara cell proliferation on matrix substrates**. Adherent cells at 18 hours were treated with BrdU in 10% FCS containing media for 24 hours. The percentage of cells incorporating BrdU over this time period on Fn, Col I, Lm 511, Col IV, Lm 332, Lm 111 or uncoated (Un, white) slides is shown. Results are expressed as mean percent of BrdU positive cells per high power field +/- SEM from three independent isolation experiments. No conditions were statistically significant from uncoated wells.

Clara cells proliferate in response to airway epithelial injury and are the progenitor cell of the small airway epithelium [5,37,38]. Alterations in the extracellular matrix during injury may regulate the Clara cell proliferative response. To examine the effects of matrix substrates on Clara cell proliferation, freshly isolated Clara cells were plated on chamber slides that were precoated with matrix proteins. After allowing cells to adhere for 18 hours, non-adherent cells were removed and the remaining Clara cells were incubated for 24 additional hours in the presence of BrdU. Since there was differential adhesion of Clara cells to matrix substrates (Fig. 2A), the number of cells incorporating BrdU was calculated as a percentage of the total number of adherent cells. All conditions demonstrated approximately 20–25% of cells incorporating BrdU (Fig. 3) without any significant difference between matrix substrates. Thus, unlike adhesion, the matrix substratum had no effect on the proliferative activity of our preparation of Clara cells.

### Clara Cells Demonstrate Differential Migration to Matrix Substrates

Since extracellular matrix molecules can direct migration of both airway ciliated and alveolar type II epithelial cells [12,14], the effects of matrix gradients on Clara cell migration were examined. Although all matrix components examined had some chemokinetic activity, checkerboard analyses revealed that none demonstrated significant directional chemotaxis (data not shown). Therefore, freshly isolated Clara cells were tested for haptotactic migration in Boyden chambers with membranes coated on the undersurface with matrix substrates for 24 hours.

**Figure 4:**
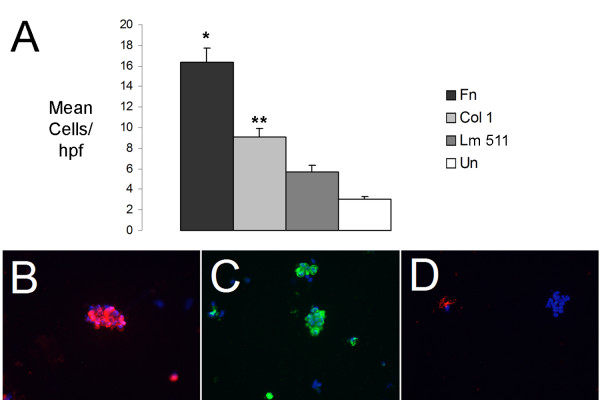
**Clara cell haptotaxis toward matrix peptides**. **(A) **Migration to the lower membrane surface was measured per high power field to membranes undercoated with Fn, Col I, Lm 511 or uncoated control (Un, white) membranes. Results are expressed as mean number of cells per high power field +/- SEM from three independent isolations. p values for comparison with uncoated control. *p < 0.001, **p < 0.05. Representative example of migrated cells on the undersurface of a Fn coated membrane stained for **(B) **epithelial cytokeratins (red) stain, **(C) **CC10 (green) and **(D) **β-tubulin (red) with DAPI nuclear counterstain.

The Clara cells migrated toward the adhesive gradient in the substratum in all conditions (Fig. 4A). There was little migration without a haptotactic gradient, so differences were significant despite relatively small numbers of migrated cells. The pro-migratory effect of the Fn haptotactic gradient (16 +/- 1.3) was greater than that of the other substrates (p < 0.03 compared to Col I, p < 0.01 compared with Lm 511) similar to the adhesive effects of Fn on the Clara cells. Col I (9 +/- 0.8), which had the capacity to promote Clara cell adhesion (Fig. 2A), also increased haptotaxis over uncoated membranes. Lm 511 (5.6 +/- 0.7) demonstrated only a non-significant trend toward increased haptotaxis over the uncoated control membrane. Lm 111 and Col IV did not demonstrate any significant haptotactic activity (data not shown).

Adhesion of Clara cells to membranes prepared for haptotaxis (coating of undersurface only) was also examined to ensure migration effects were not due to adhesion differences. Although there is a more rapid adhesion and spreading on Fn undercoated membranes, by 4 hours there was no significant difference in the number of cells adherent to the upper surface of membranes in any of the conditions examined (data not shown).

To establish that the migratory cells were Clara cells and not the contaminating ciliated or alveolar type II epithelial cells, the cells on the underside of the membrane were stained for CC10, epithelial pan-cytokeratin, β-tubulin and SP-C. Nearly all of the migrated cells were positive for epithelial cytokeratins (90%) (Fig. 4B) and the majority were CC10 expressing (50%) (Fig. 4C). Most β-tubulin is shed when ciliated cells spread, and we found only rare cells positive for β-tubulin (Fig. 4D) or SP-C (data not shown). Thus the majority of migrated cells were Clara cells. Likely the downregulation of CC10 production is responsible for some of the decrease in percentage of CC10 positive cells applied versus migrated, but the contribution of dedifferentiated ciliated cells can not be excluded.

### C22 Cells Migrate like Clara Cells to Matrix Proteins

Our modified isolation procedure has provided a reliable method for obtaining a relatively pure population of Clara cells, but the yield is too low for some studies and primary Clara cells still require serum for adhesion which contains Fn. Since we can not generate completely pure Clara cell preparations, we repeated the migration assays with the C22 line to evaluate if they were a valid surrogate for Clara cell migratory activities.

**Figure 5:**
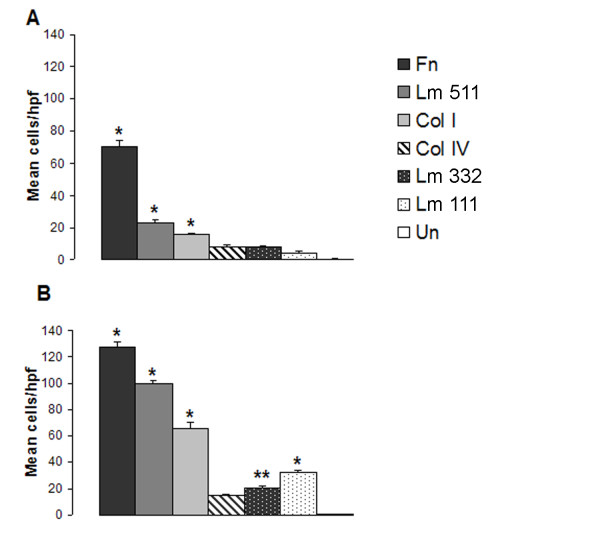
**C22 haptotaxis toward matrix peptides**. Migration to the lower membrane surface after 8 **(A) **and 16 hours **(B) **was measured to Fn, Lm 511, Col I, Col IV, Lm 332, Lm 111 or uncoated control (Un). Results are expressed as mean number of cells per high power field +/- SEM from five independent experiments. p values for comparison with uncoated control. *p < 0.001, **p < 0.05.

C22 cells were assayed at 8 and 16 hours because of the greater migratory activity of this cell line compared with Clara cells. Although the 8 hour data demonstrated significant migration towards Fn (Fig. 5A), the 16 hour time point was utilized for subsequent experiments because additional substrates demonstrated significant haptotactic activity at this time point (Fig. 5B). Fn elicited the greatest migratory response (128 +/- 3 migrated cells per hpf at 16 hours, p < 0.001 compared with all substrates). Lm 511 (99 +/- 3) and Col I (65 +/- 5) also demonstrated significant haptotactic activity at 16 hours (significantly different from each other p < 0.001 and control p < 0.001). Lm 111 was less pro-migratory (31 +/- 2, p < 0.001), but more efficient than Lm 332 (20 +/- 2, p = 0.002) and Col IV (15 +/- 1.5, p = 0.091) (all p values listed for comparison with control and not significantly different from each other). Differences seen in the total number of migrated cells between the Clara (Fig. 4A) and C22 cells (Fig. 5) likely represents enhanced adhesion of C22 cells. In addition, the C22 cells did not require serum in the upper chamber, which contains some Fn that will alter the migratory gradient in the primary Clara cell assays.

### C22 Cell Migration Is Predominantly RGD Integrin-Dependent

**Figure 6:**
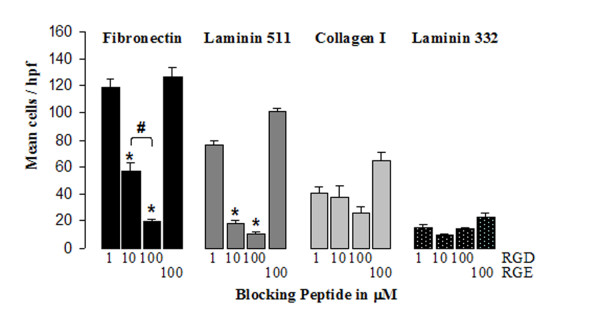
**C22 haptotaxis after pretreatment with integrin blocking peptides**. C22 cells were pretreated with GSRGD peptide (100, 10 or 1 μM) or GSRGE peptide (100 μM), then the number of migrated cells at 16 hours was measured. Independent experiments were performed for Fn (black), Lm 511 (dark grey), Col I (light grey) or Lm 332 (black speckled) undercoated membranes. Results are expressed as mean number of cells per high power field +/- SEM from three independent experiments. *p < 0.001 compared with DMEM control, ^#^p < 0.05 compared with 10 μM dose.

Preincubation of C22 cells with integrin blocking (GSRGD) and irrelevant (GSRGE) peptides was performed to determine the mechanism of C22 cell interaction with the matrix substrates. Increasing concentrations of the RGD-containing peptide resulted in a dose-dependant inhibition of C22 cell haptotaxis to Fn (118 +/- 7, 56 +/- 6, 19 +/- 2) (Fig. 6) as compared to the absence of peptide (128 +/- 3; Fig 5B). Haptotaxis to Lm 511 was also significantly inhibited by the RGD containing-peptide (76 +/- 4, 18 +/- 2, 10 +/- 2 vs. 99 +/- 3), but the haptotaxis to Lm 511 did not demonstrate the same dose response as was seen with Fn, indicating the migration to Lm 511 was more sensitive to RGD dependent integrin blockade than Fn. This is interesting because intact Lm 511 is not known to interact with RGD specific integrins. However the minimally pepsin digested placental laminin (which is actually a mixture of Lm 511 and 521) used in this assay has been described to have adhesive activity that is enhanced with further pepsin digestion, suggesting a cryptic RGD site, similar to that described in Lm 111, may be present in this preparation [39].

Although there appeared to be a trend towards decreased haptotaxis to Col I after treatment with the RGD peptide, this was not significant due to a relatively high variation in C22 haptotaxis to Col I (40 +/- 5, 38 +/- 7, 25 +/- 5 vs. 31 +/- 2).

Lm 332 (13.8 +/- 2, 9.4 +/- 1, 14.9 +/- 3), which is another common component of airway epithelial basement membrane, was less of a pro-migratory substrate for C22 cells (20 +/- 2; Fig. 5), and demonstrated no significant inhibition of migration when pre-incubation with RGD was performed (Fig. 6). The RGE-containing peptide did not alter the migrational capacity of C22 cells to any matrix substrate (No difference when compared with Fig. 5B).

## Discussion

In this paper, we describe a reproducible procedure for murine Clara cell isolation, evaluate Clara cell migration to several matrix substrates relevant to wound repair, and characterize the immortalized Clara-like cell line (C22), as a possible surrogate for Clara cells in migration assays. This work lays a foundation for future studies regarding the Clara cell in injury models where migration from a protected cell niche across matrix that may be altered after injury is present. The role of the Clara cell in small airway repair makes this information integral to our understanding of lung injury and repair.

Our modified technique of mouse Clara cell isolation provides relatively pure yields of Clara cells without the need for elutriation, but Clara cell lines are needed to study Clara cells because clumping of this population with ciliated and/or type II cells results in an inability to remove non-Clara cells without significantly decreasing yields. All of the airway epithelial cell type specific markers are lost with time in cultures as the lung epithelial cells de-differentiate. The inability to monitor airway epithelial cell type in culture makes the Clara-like C22 cell line essential to confirm our findings are not skewed by contaminating ciliated cells. Mature Clara cells can also be developed in air-liquid interface cultures, but this method produces mostly ciliated cells and is more representative of a mouse trachea [40,41].

The preferential interaction of Clara cells to Fn and Col I *in vitro *suggests that the provisional matrix seen in epithelial injury plays an important role in promoting Clara cell migration *in vivo*. Col I and Fn are not present in the epithelial basement membrane, but can be major components of the provisional matrix seen after injury [42]. Uninjured basement membranes of adult terminal airway epithelial cells usually contain Lm 332 and Lm 511, as well as Col IV [36,43-46]. It is known that degradation of the matrix can reveal cryptic sites with pro-migratory activity, so the pepsinized Lm 511 used in these experiments may also represent an injured basement membrane rather than a stable mature basement membrane [10,47]. The migratory effect of Lm 511 in C22 cells was significantly RGD-dependent, suggesting that a previously described cryptic RGD site is present in this preparation, but not in Lm 332 or intact Lm 111 [39]. This is consistent with the findings of others that the commercial preparation of Lm 511 contains activities that are not present in intact basement membrane [48]. Of note the cryptic RGD site of Lm 111 was found to bind β3 but not β1 integrins in osteoclasts, suggesting that αvβ3 (the classic vitronectin binding integrin) is involved [49]. However, consistent with published reports [19], we have been unable to detect β3 integrin by immunohistochemistry in our Clara or C22 cells (unpublished observations), suggesting an integrin other than αvβ3 must be responsible for this activity in C22 cells.

Although Lm 332 is the most abundant laminin in the airway basement membrane, C22 cells demonstrated less haptotactic migration towards this substrate than others. It is well known that processing of both the α3 and γ2 chains of the Lm 332 trimer results in enucleation of the stationary hemidesmosomes or pro-migratory activity, respectively [10]. The Lm 332 preparation that we used should contain predominantly trimers with α3 in an anti-migratory, pro-hemidesmosomal state so the lack of pro-migratory activity of our Lm 332 preparation when compared with pepsinized Lm 511 is not unexpected.

Our use of 804 G conditioned media as a source of Lm 332 is similar to other studies [50], but the concentration of Lm 332 on the membrane is unknown and could be different than the other matrix substrates in this study. Since 804 G cells are tumour cells and express some Fn caution should be used in interpretation of the role of Lm 332 in C22 migration. However, since RGD did not block the Lm 332 pro-migratory activity, contaminating Fn was not the only source of promigratory effects in our assay.

The repair of isolated and *in vitro *differentiated bronchiolar epithelial cells requires integrin switching during migration over the provisional matrix [18]. The switch predominantly favours provisional matrix (Fn and/or vitronectin) binding integrins (α_5 _and α_v_), over the collagen and laminin binding integrins (α_2 _and α_3_) that tend to be present in uninjured epithelial cells [18]. Mouse Clara cells produce both α_5 _and α_v _integrins, both *in vivo *and *in vitro *after isolation [19]. Our data demonstrates that Fn and likely the RGD binding α_5β1 _and/or α_v_-containing integrins play a role in Clara cell migration. These results are consistent with the published data on both alveolar type II and bronchiolar ciliated epithelial cells [9,12,14]. Although both adhesion and migration was greatest to Fn, not all of the matrix substances had the same effect on adhesion and haptotactic migration. The lack of significant C22 adhesion to Col I or Lm 511, but enhanced migration to these substrates, suggests that C22 cells have receptors (likely integrin) that detect Col I and Lm 511, but signalling for adhesion and migratory responses differ.

Inhibition of C22 haptotaxis to Lm 511 by RGD was significant, consistent with a cryptic RGD site being present in this preparation of Lm 511 but not in intact Lm 111 [49]. As would be expected, inhibition of haptotaxis to Col I was not significantly altered by the RGD peptide. However Col I assays had the greatest variability, and a non-significant trend for RGD activity was present in C22 haptotaxis assays. The variability of the C22 cell line response to Col I was unrelated to passage number or confluence state prior to experiments. True inhibition of C22 haptotaxis to Col I with the RGD peptide would be unexpected, as collagen and laminin binding integrins (α_2 _and α_3_) are not RGD specific, but there could be some binding of RGD peptides to these integrins at the highest concentrations used in these experiments.

We utilized RGD activity in the absence of RGE activity to confirm some of our results are integrin dependent, but specific integrin interactions will require blocking antibodies to different integrin chains. Integrin blocking antibodies have been developed for human integrins, but the efficacy of these antibodies on mouse integrins will need to be established before further claims regarding the specific integrin matrix interaction in mouse Clara cells. Unfortunately human Clara cells are less abundant and could not be evaluated.

The provisional matrix should be a strong stimulus for airway epithelial cell migration and there has been a consistent effect of matrix substratum in both bronchiolar epithelial cells and alveolar epithelial cells, so the response of Clara cells is not surprising. It is known that bronchiolar epithelial cell migration to Fn is enhanced by exogenous epidermal growth factor (EGF) or insulin, and inhibited by toxic insults like smoke extract [14,51]. Preliminary results indicate that Clara cell haptotaxis to Fn is not affected by EGF stimulation (data not shown). Clara cells make EGF receptors as the development of mucus metaplasia in Clara-like cells requires EGF mediated signalling [52], but the role of EGF receptor signalling in Clara cells may differ from that seen in ciliated cells which demonstrate an EGF dependent increase in haptotaxis.

Of note, matrix type did not alter the rate of proliferation of primary Clara cells as long as adhesion was present. Similar to mouse bronchiolar epithelial cells and unlike mouse type II cells, significant proliferation of Clara cells occurs in culture [28,40]. It is our experience that proliferation of Clara cells occurs even in low serum conditions without exogenous growth factors, a characteristic not typical of ciliated cells [40]. As mentioned previously, the Clara cells are the small airway epithelial stem cell with the ability to differentiate into both Clara and ciliated cells and therefore may be more capable of proliferation ex vivo.

C22 cells are the only murine cell line that synthesizes all three surfactant proteins known to be products of Clara cells (A, B, and D, but not C) as well as CC10 [27]. In addition C22 cells will develop contact inhibition, form epithelial tight junctions, and a transepithelial resistance of 196–368 Ω*cm^2 ^when plated on Col IV coated membranes [27]. After we determined C22 cells are similar to Clara cells in haptotactic migration we utilized C22 cells to study the role of integrins in matrix modulation of Clara cell migration. Based on our preliminary studies, these cells provide a useful reagent, as they appear to act similar to primary Clara cells in our assays of migration. Future evaluations utilizing C22 cells should increase our knowledge of the Clara cell in small airway specific repair.

## Conclusion

In summary Clara cells can be isolated at relatively pure yields from mice without the need for an elutriator. Similar to the neighboring ciliated and type II cells, Clara cells migrate preferentially towards matrix molecules in the absence of a growth factor gradient. The provisional matrix provides adhesion and migration specific signals for Clara cells, but does not alter the proliferative responses. C22 cells are a valuable surrogate for primary Clara cells in migration assays, and should be useful in future studies of the response of Clara cells to injury.

## Competing interests

The author(s) declare that they have no competing interests.

## Authors' contributions

JJA was involved in the conception and design of the study, development of the Clara cell isolation procedure, and prepared the first draft manuscript.

DGK performed all haptotaxis experiments and primary Clara cell isolations.

TAK performed all proliferation experiments, was involved in the design and interpretation of the adhesion and proliferation experiments and critically revised the manuscript.

DdM provided the C22 cell line and advised on usage and culture of the C22 cells.

RMS was involved in the conception and design of the study, interpretation of all experiments and wrote and edited the final draft of the manuscript.

All authors read and approved the final version of this manuscript.

## Supplementary Material

Additional file 1 NBT activity and expression of epithelial cytokeratin in Clara cell preparations and mouse lavage macrophages. Freshly isolated mouse Clara cells stain (purple cytoplasmic, arrowhead) intensely for NBT with methyl green nuclear counterstain visible in non-Clara cells (arrow) (A). Clara cell preparations contain ~90% epithelial cells which stain for epithelial cytokeratin (red cytoplasmic stain in almost all DAPI positive cells) (B), while macrophages from a lung lavage demonstrate no activation of the NBT substrate (C) and do not express epithelial cytokeratins (lack of red cytoplasmic stain in DAPI positive cells) (D). Click here for file
