# Supplementary figures and images for: Clara cell adhesion and migration to extracellular matrix
Source: Respir Res. 2008 Jan 7;9(1):1. doi: 10.1186/1465-9921-9-1 (PMC2249579; doi:10.1186/1465-9921-9-1)

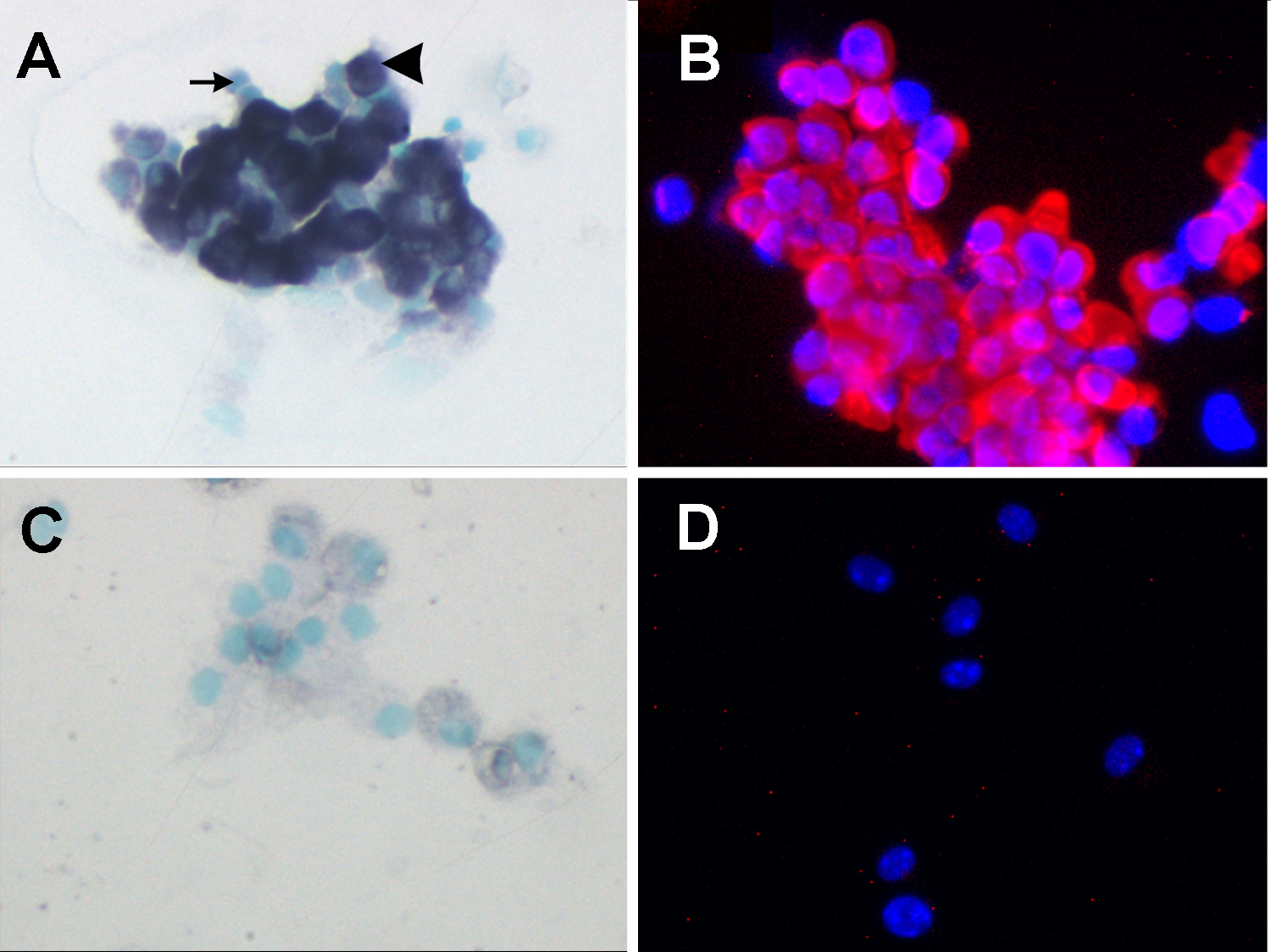

Supplement: Additional file 1 — NBT activity and expression of epithelial cytokeratin in Clara cell preparations and mouse lavage macrophages. Freshly isolated mouse Clara cells stain (purple cytoplasmic, arrowhead) intensely for NBT with methyl green nuclear counterstain visible in non-Clara cells (arrow) (A). Clara cell preparations contain ~90% epithelial cells which stain for epithelial cytokeratin (red cytoplasmic stain in almost all DAPI positive cells) (B), while macrophages from a lung lavage demonstrate no activation of the NBT substrate (C) and do not express epithelial cytokeratins (lack of red cytoplasmic stain in DAPI positive cells) (D). [file 1465-9921-9-1-S1.tiff]
